# Chicoric Acid and Chlorogenic Acid: Two Hydroxycinnamic Acids Modulate the Glucose 6-Phosphatase Activities in Pancreatic INS1 Beta-Cells—Novel Data in Favor of Two Putative Conformations of the G6Pase Within the ER Membrane

**DOI:** 10.3390/molecules30193949

**Published:** 2025-10-01

**Authors:** Didier Tousch, Melodie Thomasset, Karine Ferrare, Anne-Dominique Lajoix, Jacqueline Azay-Milhau, Patrick Poucheret

**Affiliations:** 1 Qualisud, University Montpellier, CIRAD, Institut Agro, Avignon Université, University de La Réunion, CEDEX 5, 34095 Montpellier, France; karine.ferrare@umontpellier.fr (K.F.); patrick.poucheret@umontpellier.fr (P.P.); 2 EA 7288, Biocommunication in Cardio-Metabolism, University of Montpellier, 15 Avenue Charles Flahault, BP 14491, CEDEX 5, 34093 Montpellier, Franceanne-dominique.lajoix@umontpellier.fr (A.-D.L.); jacqueline.milhau@umontpellier.fr (J.A.-M.)

**Keywords:** glucose-6-phosphatase, chicoric acid, chlorogenic acid, hydroxycinnamic acids, hepatocytes, endocrine pancreatic insulin secreting cells, metabolic syndrome, health

## Abstract

Chicoric and chlorogenic acids (CRA and CGA), two caffeic acid derivatives found in a large variety of plants, particularly in Asteraceae, are known to modulate glucose-6-phosphatase (G6Pase) in hepatic and muscle cells. The aim of the present study is to use CRA/CGA to explore the modulation role and molecular mechanism of endocrine pancreatic beta-cells’ insulin secretion. The G6Pase enzyme activity influenced by caffeic and derivatives alone or in combination was assessed on microsomal fractions of INS1-beta-cells and hepatocytes. Overall, our results show inverse effects of CGA/CRA, allowing us to investigate the G6Pase activity modulation under low and high glucose concentrations. Our data strongly suggests the existence of two putative forms of the G6Pase enzyme. Based on these observations, we formulate the hypothesis of an adaptative bi-conformational model of G6Pase enzyme activity modulation depending on the level of the beta-cell glucose exposure.

## 1. Introduction

Chlorogenic acid (CGA) and chicoric acid (CRA), a monocaffeic quinic acid and a dicaffeic tartaric acid, respectively, belong to the hydroxycinnamic acid compound family. They are produced in a large variety of plants, especially in the Asteraceae family [1,2]. Plants producing CGA and/or CRA contain complex combinations of a wide range of caffeoyl acid derivatives [2,3]. While CGA is produced in various Asteraceae species, CRA is produced only in some of them, such as *Echinacea purpurea* or *Cichorium intybus* [4,5]. The interests of these two antioxidant phytochemicals lay in their pleiotropic effects [5,6,7,8,9]. CGA and CRA exhibit antibacterial, cardioprotective, and anti-carcinogenic activities as well as hypoglycemic and hypolipidemic effects [5,6,7,8,9]. The bioactive effects of CGA and CRA are often described as related to their antioxidant properties [8,9,10,11,12,13].

Many CGA and CRA natural extracts have shown in vivo antidiabetic properties [10,14,15]. In vitro cellular tests showed the ability of NCRAE (Natural Chicoric Acid Extract)/CRA natural *Cichorium intybus*-root extract to (i) promote insulin secretion by pancreatic beta-cells and (ii) increase the insulin sensitivity as well as the glucose uptake in muscle cells [16,17]. A majority of extracts, due to their natural origin and type of extraction process, are always particular in terms of qualitative and quantitative combinations of compounds [10,14,18]. For example, the chemical analysis of NCRAE/CRA extracts [10], also described by Tousch et al. [16], has shown a relative content of 80 to 95% CRA combined with 5 to 20% CGA. This data suggests that the observed effects could be attributed to the specific quantitative combination of CGA and CRA in the extracts mentioned. The impact of CRA alone on the in vitro and in vivo effects previously described is difficult to assert in this context. The difficulty in purifying one single bioactive compound from crude plant extracts is discussed by many articles [19,20,21]. Despite this factual difficulty, results obtained with CGA and CRA extracts showed some divergences in their effects. When extracts were tested on isolated pancreatic Langerhans islets, a 90% CGA extract promoted non-glucose-dependent insulin secretion, while an NCRAE/CRA extract promoted glucose-dependent insulin secretion [16]. CGA was reported to induce a decrease in (i) glucose intestinal absorption and (ii) hepatic glucose release [8]. Hemmerle et al. [22] demonstrated how CGA interacts with the translocase T1 of the G6Pase complex [23] to inhibit G6P uptake into the endoplasmic reticulum (ER). In the hepatic tissue, G6Pase catalyzes the terminal step of the glycogenolytic and gluconeogenic pathways, converting G6P into glucose. The T1 transporter translocates G6P into the ER lumen [23]. CGA blocks T1 translocase, preventing G6P from entering the ER, which results in a decrease in G6Pase activity as a result of a lack of substrates. The lowering of dephosphorylated glucose levels causes a decrease in the release of glucose by hepatic cells, contributing to decreased glycemic control [24,25]. Paradoxically, glucose induces G6PC gene coding for the catalytic G6Pase domain [24,25]. The G6PC gene which is expressed in hepatic tissue, is one of the three genes (G6PC, G6PC2, and G6PC3) encoded for G6PC catalytic subunits [26]. The G6PC gene expression deficiency in the glycogen storage disease type Ia is responsible for an overaccumulation of G6P in hepatic cells [27]. In this context, it was shown that CGA treatments in vivo in mice underlie glycogen storage disease type Ib [28].

The levels of G6Pase activity in pancreatic tissue have been the subject of controversy for some time. Consequently, it was unclear whether G6Pase played a role in this tissue [29,30,31,32]. The general consensus is that G6Pase is present and functional in pancreatic beta-cells [32,33,34]. Due to the difficulty in detecting significant G6Pase activity in pancreatic tissue for many years, it has been considered that labile G6Pase activity constitutes a ‘futile cycle’ that modulates the glucose metabolic flux [35]. Initially named the islet-specific G6PC-related protein (IGRP) gene, it is admitted that the G6PC2 gene is expressed in beta-cells [33,34] and is involved in modulating fasting blood glucose levels in vivo [33,35]. Previous studies suggested the possible role or connection of G6Pase in the regulation of glucose-sensitive insulin secretion (GSIS) [32,33]. A G6PC2 gene knockout (KO) in mice led to a leftward shift in the dose–response curve for glucose-stimulated insulin secretion, suggesting that G6PC2 negatively regulates glucose-stimulated insulin secretion and abolishes glucose cycling [34]. A CRISPR/Cas9 G6PC2 KO in a mouse TC3 beta-cell line showed that G6PC2 regulates GSIS through the (i) modulation of glycolysis, (ii) reduction in the potential redox in cells, and, also, (iii) strengthening of mitochondria metabolism [36]. More recently, SNP (Single Nucleotide Polymorphism) studies on the G6PC2 gene unraveled the modulation of G6Pase enzyme activity. According to the authors, G6Pase modulation is linked to a structural modification of the ER membrane surrounding the G6Pase complex [37]. The data suggests that pancreatic G6Pase plays a subtle role in regulating glucose-induced insulin secretion. Despite this, its dysfunction does not cause any open pathology, but it could lead to the development of type 2 diabetes. In some animal models of type 2 diabetes, a loss of glucose-stimulated insulin secretion is correlated with an enhancement of the glucose cycle and increased G6Pase activity [38,39]. Iizuka et al. [39] showed that the modulation of glucose-stimulated insulin release can be obtained by altering glucose cycling through the elevation of G6Pase activity. However, an insulin secretion deficiency is not exclusive to the enhanced level of G6Pase activity found in diabetic rodent islets of Langerhans [39]. Indeed, Fulceri et al. have shown that exacerbated G6Pase activity in the INS1 beta-cell line is associated with a possible role in modulating insulin secretion [40].

Regarding the literature data, the biological role of pancreatic G6Pase needs to be better understood. Although the KO genetic approach has already made significant progress, it is still necessary to conduct investigations to fully understand the true impact of the proposed ‘G6Pase futile cycle’ on the regulation of the glucose metabolic flux. For this purpose, CRA and CGA, which modulate G6Pase, seem to be of interest. For instance, CGA, known as a hepatic G6Pase inhibitor, should be of interest to be tested on pancreatic beta-cells. Since (i) the genetic suppression of G6PC2 in pancreatic beta-cells induced an increase in glucose-stimulated insulin secretion, and (ii) CGA is a stimulator of insulin secretion but its effect is not glucose-dependent [16], it therefore appears of importance to test the putative inhibitor effect of CGA. The ability of CRA to modulate liver enzyme activity has not been evaluated, and the same is true for pancreatic enzymes. These elements led to the aims of the present study: (i) to explore the CGA and/or CRA modulation of G6Pase enzymes independently and in combination to detect putative synergy as well as (ii) to test natural extracts containing CGA and CRA to compare their biological effects to the results obtained with isolated compounds. Our goal is to assess how CRA and CGA affect G6Pase activity to provide insight into the role of the enzyme in the pancreatic cell.

## 2. Results

### 2.1. Glucose Release Under Glucagon Stimulation and Microsomal G6Pase Activity in Hepatic Cells in Presence of CGA and CGA

**Figure 1:**
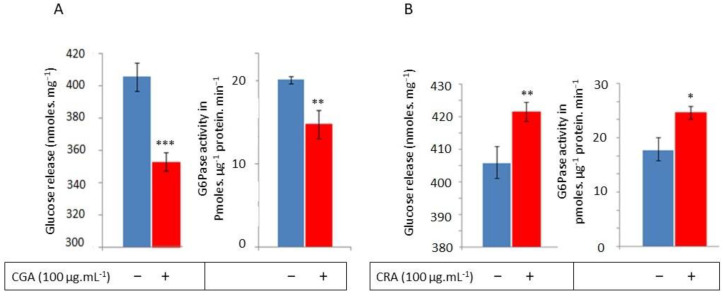
CGA and CRA effects on hepatic cells: Glucose release and G6Pase activity evaluations. CGA (**A**) and CRA (**B**) effects on the glucose release by hepatic cells under glucagon stimulation is expressed in nmoles·mg^−1^ protein and on the G6Pase activity of hepatic microsomes in pmoles Pi·µg^−1^·protein·min^−1^. The assays noted as (−) are the controls, while the assays noted as (+) are with CGA or CRA added. The results are the mean of six assays (two independent enzymatic assays by three independent experiments; *n*= 6) with standard error bars (*p* < 0.05 *, *p* < 0.01 **, *p* < 0.001 ***).

First, we studied CGA’s and CRA’s effects on the liver. Hepatic primo-culture cells were grown in a medium enriched by CGA or CRA at 100 µg·mL^−1^. Cells were submitted to glucagon stimulation to enhance glucose release. The presence of CGA in the medium at 100 µg·mL^−1^ induced a 13.5% reduction in glucose release (Figure 1A) in accordance with the literature [13].

Second, we used commercial hepatic microsomes that we submitted to a G6Pase activity assay in the presence of CGA (100 µg·mL^−1^) or not (Control). A significant decrease in the G6Pase activity was observed from 30 ± 2 pmoles Pi·min^−1^·µg^−1^ protein in the control to 22 ± 6 pmoles Pi·min^−1^·µg^−1^ protein with CGA (Figure 1A). In accordance with previously published data, our results allowed us to validate our experimental procedure [10,17,18].

In contrast, CRA (100 µg·mL^−1^) led to a slight significant stimulation of glucose release (around 5%) by hepatic cells and increased the hepatic G6Pase microsomal activity at 26 ± 5 pmoles Pi·min^−1^·µg^−1^ protein (versus 36 ± 3 in control) (Figure 1B). According to this finding, CRA has a clear activator effect on G6Pase and leads to an increase in glucose release from hepatic cells under glucagon stimulation. The opposing action of CRA and CGA on hepatic G6Pase activity is correlated with the action of these two substances on glucose release from hepatic cells. This concomitance of CRA and CGA effects clearly demonstrates the involvement of hepatic G6Pase in the regulation of glucose availability by the liver tissue.

### 2.2. Microsomal G6Pase Activities from INS1 Beta-Cells Cultivated in RPMI with Different Glucose Concentrations

**Figure 2:**
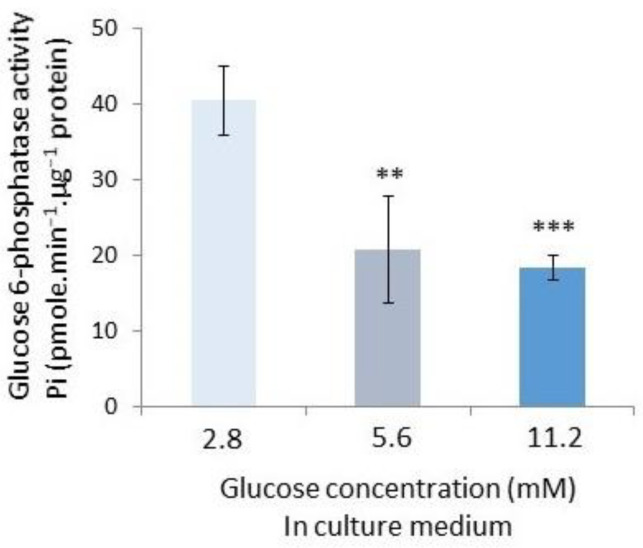
G6Pase activities in INS-1 beta-cells growth in RPMI medium containing three concentrations of glucose (2.8, 5.6, and 11.2 mM). The G6Pase activity is expressed in pmoles Pi·µg^−1^·protein·min^−1^. The results are the means of six assays (two independent enzymatic assays by three independent experiments; *n* = 6) with standard error bars (*p* < 0.01 **, *p* < 0.001 ***).

As already mentioned in the introduction, the INS-1 beta-cell line has exacerbated G6Pase activity [40]. This explains why we chose this cell line as a model for G6Pase activity measurements. In this experiment, we evaluated the G6Pase activities under different concentrations of glucose. For the growth of INS-1 beta-cells, the medium was supplemented with three glucose concentrations (2.8, 5.6, and 11.2 mM), which correspond to hypoglycemia, normoglycemia, and hyperglycemia in vivo. After separating the microsomal fraction of cells, we examined the activity of G6Pase at three glucose concentrations. The glucose concentration has an impact on G6Pase activity, as demonstrated in Figure 2. The activity of INS1 G6Pase decreased as the glucose in the medium increased. We have evaluated the G6Pase activity value at 40.5 ± 3.0 Pi·pmole·min^−1^·µg^−1^ under low glucose levels (2.8 mM) and at 18.4 ± 1.0 Pi·pmole·min^−1^·µg^−1^ under high glucose levels (11.2 mM). In a middle glucose concentration of 5.6 mM, the value at 20.9 ± 3.5 Pi·pmole·min^−1^·µg^−1^ led to a value close to 11.2 mM glucose. According to our findings, the G6Pase activity is significantly influenced by the glucose concentration in the culture medium of INS-1 beta-cells. Cells have a high potential for G6Pas activity when glucose concentrations are low. At higher concentrations, at least from 5.6 mM, the potential for G6Pase activity is lower.

### 2.3. CGA and CRA Effects on the Microsomal G6Pase Activities from INS1 Beta-Cells Cultivated in RPMI Medium (11.2 mM Glucose)

**Figure 3:**
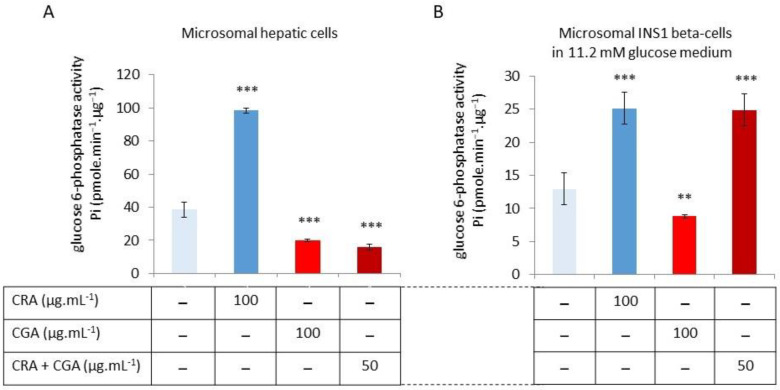
G6Pase activities of microsomes isolated from hepatic (**A**) or INS-1 beta-cells (**B**) growing in standard mediums. Enzymatic reactions have been carried out with or without CGA or CRA at 100 µg·mL^−1^ in enzyme buffer and also with a mix of CGA and CRA at 50 µg·mL^−1^ each. The G6Pase activity is presented in pmoles Pi·µg^−1^·protein·min^−1^. The data are expressed as means of *n*= 6 (two independent enzymatic assays for each of the three independent experiments) with standard error bars (*p* < 0.01 **, *p* < 0.001 ***).

The glucose sensitivity of the pancreatic G6Pase cells requires studying CGA and CRA effects in the three glucose concentrations. As the initial step, we examined the capacity of the two natural substances to regulate pancreatic G6Pase activity. In a prerequisite experiment, we extracted microsomes from INS1-1 beta-cells grown on a standard RPMI medium at 11.2 mM glucose. The first experiment was carried out in parallel with hepatic microsomes, which will be used as quality controls for natural substances. Results in Figure 3A indicate that CGA leads to the inhibition of hepatic G6Pase activity (19.9 ± 0.8 Pi·µg^−1^·protein·min^−1^ versus 38.6 ± 1.5 in the control), while CRA induced a stimulation of the G6pase (98.4 ± 1 Pi·µg^−1^·protein·min^−1^), which is in accordance with the previous findings (Figure 1). An experiment involving a CGA/CRA mixture (50:50) resulted in an inhibition of G6Pase activity that was comparable to that of CGA (15.9 ± 0.8 Pi·µg^−1^·protein·min^−1^). The result could be interpreted as a dominant effect of CGA because it inhibits translocase T1, which is responsible for limiting the G6P uptake in the ER lumen, the substrate of G6Pase; CGA and CRA were demonstrated to be bioactive. In Figure 3B, we show the G6Pase activity of INS-1 beta-cell microsomes exposed to CGA and CRA. Hepatic cells show a higher G6Pase activity than INS-1 cells, respectively, at 38.6 ± 1.5 Pi·µg^−1^·protein·min^−1^ and 13 ± 2.5 Pi·µg^−1^·protein·min^−1^. In the INS-1 beta-cells, CGA (100 mg·mL^−1^) has greatly inhibited the G6Pase activity by 36% (8.8 ± 1.0 Pi·µg^−1^·protein·min^−1^), whereas CRA (100 µg·mL^−1^) has induced a twofold increase (25.1 ± 2.5 Pi·µg^−1^·protein·min^−1^). The G6Pase complex found in INS-1 beta-cells is equally sensitive to CGA and CRA as hepatic cells, according to our findings. In the CGA/CRA mix (50:50) test, it was shown that the INS-1 beta-cell activity in the G6Pase was significantly increased (24.9 ± 2.5 Pi·µg^−1^·protein·min^−1^). The last result is remarkable because it is contrary to what was achieved on hepatic cells.

### 2.4. CGA and CRA Effects on the G6Pase Activities in INS1 Beta-Cells Cultivated in the 2.8, 5.6, and 11.2 mM Glucose in RPMI Medium

**Figure 4:**
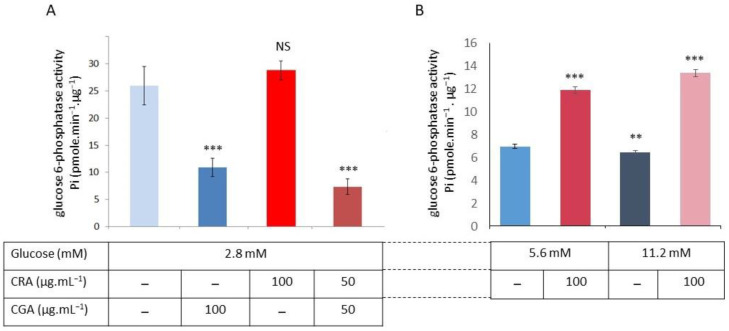
CGA and CRA effects on the G6Pase activities in INS-1 beta-cells growth at 2.8 mM glucose in RPMI medium (**A**) and at 5.6 and 11.2 mM glucose in RPMI medium (**B**). The G6Pase activity is given in pmoles Pi·µg^−1^·protein·min^−1^. The data are expressed as means of *n* = 6 (two independent enzymatic assays for each of the three independent experiments) with the standard error bars (*p* < 0.01 **, *p* < 0.001 ***) and NS (not significant).

Now that we know the effects of CGA and CRA on the G6Pase activity of INS-1 beta-cells in a medium with 11.2 mM glucose, we need to test their effects at three glucose concentrations (2.8, 5.6, and 11.2 mM). Cells were grown in a standard RPMI medium (11.2 mM) for 72 h before being replaced with RPMI containing either 2.8, 5.2, or 11.2 mM glucose. One day before the experiment, CGA or CRA and a CGA/CRA mix (50:50) was added in the cell culture mediums. After rupturing cells, microsomal fractions could be isolated and evaluated for G6Pase activity. Figure 4 confirms our previous findings (Figure 2), which demonstrated that G6Pase activity was clearly inversely correlated with the glucose level. The activity of G6Pase was three times more elevated in the 2.8 mM glucose (Figure 4A) than in the 11.2 mM glucose (Figure 4B). At a glucose concentration of 2.8 mM, CGA has an inhibitory effect, while CRA has no significant effect on G6Pase activity (10.9 ± 2 Pi·µg^−1^·protein·min^−1^ versus 25.9 ± 4.5). The CGA/CRA mix (50:50) showed a similar inhibition of CGA (Figure 4A). The G6Pase activity at both the 5.6 and 11.2 mM glucose is strongly similar (Figure 4B), as we have previously observed (Figure 2). CRA and a CGA/CRA mix (50:50) have a significant stimulating effect on the activity of G6Pase (Figure 4B).

### 2.5. CRA’s Effect on Glucose-Stimulated Insulin Secretion

**Figure 5:**
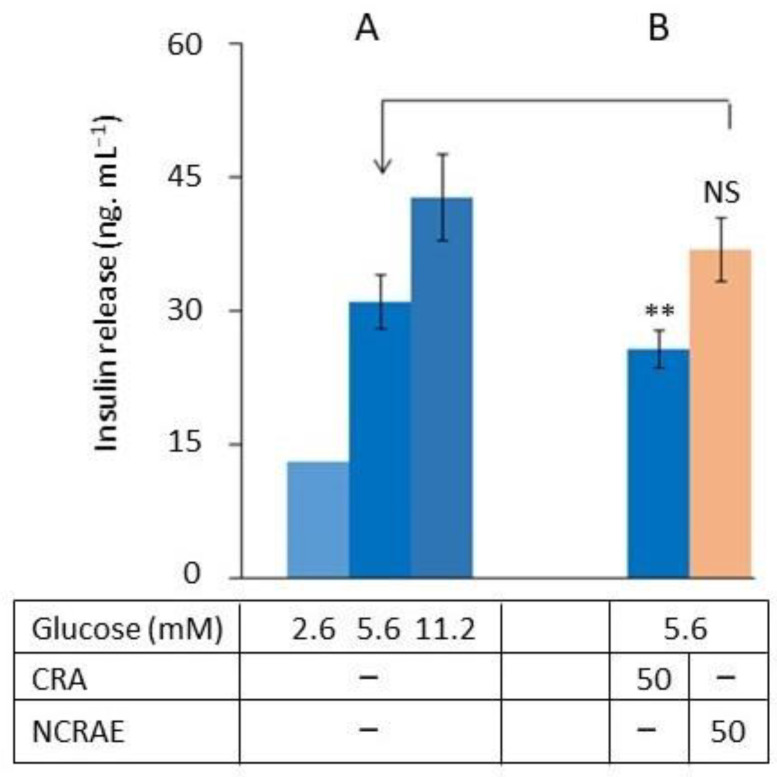
In vitro glucose-induced insulin secretion in INS-1 beta-cells. After being grown in the INS-1 beta-cells in RPMI medium, cells were placed in KRB with different glucose concentrations, and modulation of insulin secretion in medium were evaluated (**A**). Cells were placed in KRB at 5.6 mM of glucose with NCRAE natural extract added at 100 µg·mL^−1^ or CRA at 100 µg·mL^−1^, and insulin secretion were measured (**B**). The insulin release is given in ng of insulin·mL^−1^. The data are expressed as means of three independent assays (*n*= 3 with standard error bars. NS (no statistically significant difference) result. Statistically significant difference *p* < 0.01 **.

We assessed the CRA’s and NCRAE’s [10,16] effects on glucose-stimulated insulin secretion. The INS-1 beta-cells were grown in the standard RPMI medium (11.2 mM glucose) before being replaced by RPMI with different glucose concentrations (2.6, 5.6, and 11.2 mM). The glucose sensitivity of the cells (Figure 5A) is demonstrated by the increase in insulin secretion correlated with the glucose level. In the assay at 5.6 mM glucose, CRA at 100 µg·mL^−1^ provokes a decrease in the insulin secretion (Figure 5B). We were interested in NCRAE, a chicory root extract, because of its recognized ability to stimulate insulin secretion and its combined CGA/CRA composition with a 30/70 ratio [8,14]. We observed a slight increase in the insulin release in the presence of NCRAE in the medium in accordance with the literature, although this was not statistically significant (*p* = 0.099), which can be explained by the small number of repetitions (*n* = 3).

## 3. Discussion

G6Pase initiates the final stage of glycogenolysis to promote the release of glucose from the liver into the bloodstream. G6Pase is a key enzyme in glucose homeostasis, which requires a strong regulation of its activity. It represents a potential therapeutic target for treating type 2 diabetes. The inhibition of its activity may contribute to reducing chronic hyperglycemia. CGA was shown to inhibit G6Pase activity by applying it in vitro to hepatic cells under glucagon stimulation, leading to a decrease in glucose release [17,18]. In our experiment, this data from the literature was used as a validation model. Our investigation was carried out to compare (i) hepatic and insulin-secreting cells’ responses in vitro and (ii) responses to CGA and CRA compounds. We confirmed the ability of CGA to modulate the glucose release from hepatic cells concomitantly with the G6Pase activity inhibition. In the same experimental conditions, we showed that CRA is able to significantly increase the G6Pase activity (Figure 1B) that was correlated to an increase in cells’ glucose release. This result suggests that the hepatic G6Pase is a target for CRA (a hydroxy-cinnamic acid), inducing an opposite effect to that of CGA (also a hydroxy-cinnamic acid).

G6Pase activity is well-known to regulate glucose metabolism in the liver, but its role in pancreatic beta-cells is still unknown. The role of G6Pase is controversial even though some authors suggest that it could modulate insulin secretion [23]. Fulceri et al. (2000) showed increased G6Pase activity in INS-1 beta-cell lines, suggesting a potential role in insulin secretion regulation [40]. By combining all of this data with the fact that INS-1 beta-cells have a high G6Pase activity that is easily quantifiable, we have selected them for our studies. Our approach was to test the effects of CRA and CGA on pancreatic G6pase activity. Our results show that CGA and CRA have a complex pattern of modulating G6Pase activity in INS-1 beta-cells.

According to the results in Figure 1, CGA’s (100 µg·mL^−1^) inhibitor effect on hepatic cells has been confirmed [22], allowing us to validate our experimental approach. In this condition, we have shown that CRA (100 µg·mL^−1^) induced a significant increase in the hepatic G6Pase activity. As new data, we show an inverse effect between CGA and CRA on the activity of the hepatic G6Pase. With the same approach, we have explored CGA’s and CRA’s effects on G6Pase activity in INS-1 beta-cells. The glucose sensitivity of the pancreatic cells has led us to evaluate the G6Pase activities in INS-1 beta-cells under the three glucose conditions corresponding in vivo to a hypo- (2.8 mM), normo- (5.2 mM), and hyper- (11.2 mM) glycemia. With the 2.8 mM glucose in the culture medium, INS-1 beta-cells exhibit twice as much G6Pase activity as those at the 5.6 mM and 11.2 mM glucose (Figure 2). This finding confirms previous arguments that mouse islet G6Pase can be inhibited up to 50% when exposed to 10 mM glucose compared to 5 mM glucose [27].

The results in Figure 3 show that CGA and CRA have similar effects on the G6Pase activity, both on the microsomes of hepatic cells and INS-1 beta-cells grown under 11.2 mM glucose. CGA inhibits the G6Pase activity, unlike CRA, which stimulates the enzyme activity. Simultaneously adding CGA/CRA (50:50 ratio) in the enzyme buffer completely erased the stimulation effect of CRA (Figure 3A). This result suggests that there is a significant and dominant CGA effect, which is probably due to the blocking action of G6P translocase, which restricts the G6P substrate uptake into the ER lumen. The CGA/CRA (50:50 ratio) mix on the INS-1 beta-cell microsomes leads to an increase in G6Pase activity at a similar level as CRA alone (Figure 3B). This response, which is inverse to the inhibition described for hepatic G6Pase activity, suggests a loss of the dominant CGA effect.

On the basis of our previous results, we have tested CGA, CRA (100 µg·mL^−1^), and the combination CGA/CRA mix (50 µg·mL^−1^ each) on INS-1 beta-cells grown in the medium at 2.8, 5.2 mM, and 11.2 mM of glucose (Figure 4) in order to evaluate the G6Pase activities. With 2.8 mM glucose, we observed that CGA had no effect on G6Pase activity, whereas CRA and the CGA/CRA mix significantly inhibited the enzyme activity. With the 5.6 and 11.2 mM glucose, CRA (100 µg·mL^−1^) provokes a significant increase in G6Pase activity.

**Figure 6:**
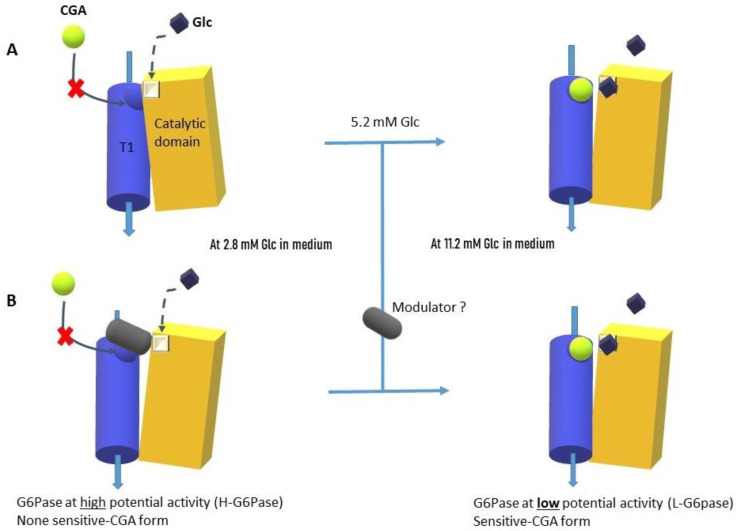
Representation of the G6Pase complex in our hypothesis of two putative forms in the RE membranes of the INS-1 beta-cells. (**A**) The H-G6Pase at low glucose concentration could switch to a L-G6Pase form between 2.8 and 11.2 mM of glucose level. (**B**) The switch can be accomplished through the hypothesis of the cytoplasmic modulator (black capsule). The modulator could be removed by glucose depending on its concentration. According to the conformational hypothesis, CGA cannot access this fixation site in the H-G6Pase form but can only do so in the L-G6Pase form. The switch between the two forms would occur around normoglycemia (5.2 mM of glucose) (vertical line).

Together, the data showed that there were two distinct responses of the G6Pase complex from the INS-1 beta-cell to CGA or CRA. So, it is possible to hypothesize that two G6Pase complexes are present on the ER membrane of the pancreatic INS-1 beta-cell: (i) one complex with high potential activity (H-G6Pase) under low glucose concentrations equivalent to hypoglycemia in vivo, and (ii) the other with low potential activity (L-G6Pase) under more elevated glucose concentrations (5.2 to 11.2 mM) equivalent to hyperglycemia in vivo (Figure 6A). Additionally, we can suggest that the supposed H-G6Pase is not responsive to CGA (H-G6Pase/CGA-NS), whereas L-G6Pase is CGA-sensitive (L-G6Pase/CGA-S). This hypothesis suggests a transition zone (between low and high glucose) from one H-G6Pase complex to another (L-G6Pase). There was less G6Pase activity with the 5.2 mM glucose in the medium than with the 2.8 mM glucose. However, CRA is able to increase the G6Pase activity, as we found in the 11.2 mM glucose medium. The data tends, therefore, to support the idea that the G6Pase complex under the 5.2 mM glucose condition could be the L-G6Pase form. Furthermore, it is possible to suggest that 5.2 mM lies in the switching zone between the two forms, the transition zone from hypoglycemia to hyperglycemia.

Furthermore, we have observed that CRA seems to have a glucose-mimicking action that is capable of reducing the G6Pase activity. CRA and a high glucose level could help facilitate the transition from the H to the L-G6Pase form. To discuss the two G6Pase form models of the G6Pase complex comprising glycolytic and T1 translocase domains into the ER membranes, we propose a cytoplasmic modulator that can bind (and closed) the H-G6Pase form (Figure 6B). CGA’s access would be blocked by the modulator. CRA has the potential to either compete for glucose (A) or destabilize and release the cytoplasmic modulator (B). At the conclusion, the modulator would be released either by CRA or glucose. Access to the CGA site would be made possible by either a form change in the G6Pase complex or the loss of the modulator.

Another hypothesis in accordance with the results could be considered by proposing an unknown G6P transporter (non-sensitive CGA), a hypothesis not retained so far, since published data shows the unequivocal dependence of G6pase activity on the G6P translocase T1 [41].

Finally, we tested CRA’s and NCRAE’s (a natural CGA/CRA mix) effects on INS-1 beta-cells grown in a 5.2 mM glucose medium. NCRAE had a slight stimulation effect (low significate statistical effect probably because of a low number of repetitions), which is in contrast to the decrease in insulin secretion caused by CRA.

These latest results will need to be confirmed with a larger number of repetitions, as the number of repetitions in this experiment was proved to be too low. However, our results, which show an antagonist effect of CGA and CRA, suggest that the CGA/CRA ratio in an extract, whether natural or not, could have different effects on insulin secretion.

## 4. Materials and Methods

### 4.1. Materials

Commercial synthetic chicoric acid (CRA; C7243 catalog number) and chlorogenic acid (CGA; C3878 catalog number) powders were purchased from Sigma-Aldrich (Munich, Germany). NCRAE is a natural plant extract produced following the protocol previously described by Ferrare et al. [10]. Dulbecco’s Modified Eagle’s Medium (DMEM; F 0415 catalog number) and Fetal calf serum (FCS; S3113 catalog number) were obtained from Biochrom (Berlin, Germany). RPMI 1640 with glucose at 2 g·L^−1^ (R4130 catalog number) or without glucose (R1383 catalog number), HEPES-buffered solution (H3375 catalog number), L-glutamine–penicillin–streptomycin solution (G6784 catalog number) are, respectively, 200 mM, 10,000 U, and 10 mg.mL^−1^, and sodium pyruvate, beta-mercaptoethanol, and BSA (O5470 catalog number) were obtained from Sigma-Aldrich. Commercial liver microsomes were purchased from BD Biosciences (Le Pont de Claix, France). Hepatocyte cells, provided by J. Azay-Milhau (Montpellier University, Montpellier, France), have been isolated using the previously described procedure [15].

### 4.2. INS-1 Beta-Cell Cultures

The use of the INS-1 beta-cell pancreatic rat insulinoma-derived cells (obtained by Prof. C.B. Wollheim, Geneva, Switzerland) was motivated by their glucose sensibility and also their high G6Pase activity described in the literature [40,42]. Cells were cultured in 24-wells plates at 2 × 10^5^ cells by well in complete RPMI 1640 with 2 g·L^−1^ (11.2 mM) glucose supplemented with 10% heat-inactivated FCS, 1 mM Na pyruvate, 50 mM beta-mercaptoethanol, 2 mM glutamine, 10 mM HEPES, 100 U·mL^−1^ penicillin, and 100 mg·mL^−1^ streptomycin at 37 °C in 5% CO_2_ during 5–6 days before the experiment (medium replenished on day 3) [41]. Depending on the experiment, two procedures were employed at this stage: (i) to evaluate the G6Pase activity, cells were washed once with complete RPMI before being replaced by complete RPMI without glucose, to which we added 2.8, 5.2, and 11.2 mM of glucose and also CGA or CRA at 100 µg·mL^−1^ or CGA/CRA mix (50:50) at 100 µg·mL^−1^ and incubated at 37 °C in 5% CO_2_ during 12 h, (ii) to evaluate the insulin secretion level, cells were washed twice and incubated for 90 min in glucose-free KRB buffer, BSA (2%). Next, cells were washed once with glucose-free KRB and BSA (2%) and then incubated for 30 min in KRB BSA (2%) with 2.6, 5.6, and 11.2 mM glucose and 5.6 mM glucose plus CRA or NCRAE at 50 µg·mL^−1^.

### 4.3. Insulin Secretion Measurements

The cell culture supernatants were collected to quantify the insulin released by FRET technology with the Insulin-Kit HTRF^®^ (Cis-Bio International, Saclay, France) according to manual procedure. The fluorescence levels were measured on an INFINITE^®^ F500 instrument (TECAN Group Ltd., Männedorf, Switzerland). Results are expressed in insulin release at ng·mL^−1^.

### 4.4. The Evaluation of the Glycogenolysis of Hepatocyte Cell Cultures

As previously described, hepatocytes were plated at a density of 830,000 cells by well onto collagen-coated 12-well plates in basal medium (DMEM containing 11.1 mM glucose, 100 U·mL^−1^ penicillin, 100 µg·mL^−1^ streptomycin) supplemented with 6% FCS [17,43]. Four hours after initial plating, the medium was changed for a basal medium supplemented with 100 nM dexamethasone, and plates were incubated at 37 °C during 24 h in 5% CO_2_. For the glycogenolysis test, the medium was replaced by a basal medium supplemented with 100 nM dexamethasone, 13.9 mM glucose, and 100 nM insulin, and plates were incubated for 20 h. Following this period, cells were washed twice with ice-cold PBS using amyloglucosidase (exo-1.4-beta-glucosidase) digest [43] before being immersed twice in pre-warmed (37 °C) buffer at pH 7.4 containing 117.6 mM NaCl, 5.4 mM KCl, 0.82 mM MgSO_4_, 1.5 mM KH_2_PO_4_, 20 mM HEPES, 9 mM NaHCO_3_, 2.25 mM CaCl_2_, and 0.1% (*w*/*v*) BSA. Finally, cells were incubated for 180 min in the last buffer with added glucagon (100 nM), with or without the compounds at 50 µg·mL^−1^. Glycogenolysis was measured as the amount of glucose released (nmole/well) by using a glucose oxidase kit from Megazyme. Results are presented as percentages of control values obtained in the presence of glucagon alone.

### 4.5. The Isolation of the INS1 Beta-Cells’ Microsomal Fraction

We used a methodology described by Villalpando et al. with few modifications [44]. A freshly cultured INS-1 beta-cell was performed as described above. A 5–6 day fresh INS-1 beta-cell culture was performed as described above, and 11.2 mM glucose was used to isolate the microsomal cell fraction. Cells were washed once in PBS before a trypsin treatment for 3 min at 37 °C. Cells were diluted in KRB buffer (10 mL) and collected in a sterile centrifugation tube. After centrifugation at 200 g for 5 min, cells were dispersed in 10 mL KRB for 120 min in cold water. After centrifugation, cells were dispersed in cold PBS buffer supplement with 1 mM PMSF and 1.5 mM EDTA. After 30 min of incubation, cells were collected by centrifugation and dispersed in 1.5 mL sucrose buffer at pH 7.4; 250 mM sucrose, 10 mM benzomidine, 0.005% DNase, 1 mM MgCl_2_, 4 mM HEPES, 4 µM PMSF, 4 µM pepstatin A (25 mg·mL^−1^), 4 µM aprotinin (10 mg·mL^−1^), 1X TABS buffer. The cell-lysates were obtained using a nitrogen cavitation bomb cell disruption chamber under 10 bars at 4 °C during 20 min. Lysate was homogenized by several passages through a syringe needle (27 G) before centrifugation at 5000× *g* during 10 min (4 °C). The supernatant was finally deposited at the top of a discontinuous sucrose gradient preliminary built layer by layer from bottom to top (2 M, 1.8 M, 1.6 M, 1.4 M, 1.2 M, 1 M, 0.8 M) in an ultra-centrifugation sterile tube. Centrifugation at 110,000× *g* (Beckman SW-40 rotor, Beckman Coulter, Brea, CA, USA) for 18 h allowed us to collect ten fractions between 0.8 and 1.2 M sucrose layers. Each fraction was tested for their glucose 6-phosphatase activity.

### 4.6. Glucose 6-Phosphatase Activity Test

The standard G6Pase assay [32,45] was conducted in 100 µL of buffer (10 mM HEPES, 0.25 M sucrose, 20 mM Tris-HCl, pH 7.4) supplemented with 20 mM of glucose 6-phosphate with a microsomal suspension volume equivalent to 2 µg or 4 µg of total proteins. Assays were incubated over 30 min at 30 °C. The reaction was stopped by addition of malachite green color reagent described by Daniele et al. [46], containing one part 4.2% ammonium molybdate and three parts 0.2% malachite green, 5 mol·L^−1^ H_2_SO_4_, and 0.1% Tween 20. After 5 min at room temperature, Pi was determined by optical density at 660 nm. The standard curve was obtained using a 1 M NaH_2_PO_4_ solution to prepare 1, 5, 10, and 20 nmole standard solutions. Results are expressed in pmoles of Pi produced during 1 min per µg of total proteins. In these conditions the effects of compounds were studied at 100 µg·mL^−1^ of each caffeoyl derivative or 50 µg·mL^−1^ each for the mix. The lipidic membrane integrity of microsomes was checked by the equivalent G6Pase assay in presence of 0.1% Triton X100, leading to membrane disruption and to elevated G6Pase activity as previously described by Murataliev and Vulfson (1986) [44]. As previously described, we used the acidification inhibitor effect (pH 5.5) of the medium to confirm the specificity of the G6Pase activity measured [46].

### 4.7. Statistics

Data are reported as mean plus or minus standard deviation to the mean (SD) from two independent enzymatic assays for each of the three replicate experiments (*n* = 6) or from three independent assays (*n* = 3) for the insulin quantification experiment. Statistical analysis was performed by Fisher’s protected least significant difference test at *p* < 0.05, using the Stat Graphics software (StatGraphics 18^®^ centurion, Statgraphics Technologies, Inc., The Plains, VA, USA).

## 5. Conclusions

In conclusion, our results argue the hypothesis of the presence of two G6Pases forms present into the ER membrane of pancreatic insulin-secreting beta-cells (INS-1 in our experiments). To provide additional evidence for this hypothesis, we need to confirm our results through more experiments and continue our investigations. As previously mentioned in the introduction, the genetic knockout method has not produced any conclusive results. To confirm this putative model, structural in situ analyses of G6Pase, i.e., in the ER membrane, are required. We are considering proximity-dependent labeling approaches or even immune-affinity techniques. Our investigation suggests that the pancreatic G6Pase enzyme could be involved in modulating glucose-induced insulin secretion in a particular context. To better understand and confirm this moderate and fleeting modulation, further investigations will be necessary to evaluate the extent and amplitude of G6Pase’s modulation ability.

## Data Availability

The original contributions presented in this study are included in the article.

## Abbreviations

The following abbreviations are used in this manuscript:

CRA	Chicoric Acid
CGA	Chlorogenic Acid
G6Pase	Glucose-6-Phosphatase
NCRAE	Natural Chicoric Acid Extract
IGRP	Islet-Specific G6PC-Related Protein
GSIS	Glucose-Sensitive Insulin Secretion
